# THE IMPACT OF LIFESTYLE BEHAVIORS ON HEALTHY AGING TRAJECTORIES: THE ATHLOS PROJECT

**DOI:** 10.1093/geroni/igz038.2943

**Published:** 2019-11-08

**Authors:** Christina Daskalopoulou, Yu-Tzu Wu, Artemis Koukounari, Graciela Muniz Terrera, Stefanos Tyrovolas, Demosthenes Panagiotakos, Martin Prince, Matthew Prina

**Affiliations:** 1 Social Epidemiology Research Group, Health Service and Population Research Department, Institute of Psychiatry, Psychology & Neuroscience, King’s College London, London, England, United Kingdom; 2 Faculty of Epidemiology and Population Health, London School of Hygiene & Tropical Medicine, London, England, United Kingdom; 3 Centre for Dementia Prevention, Centre for Clinical Brain Sciences, University of Edinburgh, Edinburgh, England, United Kingdom; 4 Parc Sanitari Sant Joan de Déu, Universitat de Barcelona, Barcelona, Catalonia, Spain; 5 Department of Nutrition and Dietetics, School of Health Science and Education, Harokopio University, Athens, Attiki, Greece; 6 Global Health Institute, King’s College London, London, England, United Kingdom

## Abstract

The number of people above 60 years old will double by 2050. There is a considerate variability in the health status of older people. The identification of the different trajectories that people follow as they grow older constitutes one of the aims of the ATHLOS project. In the current study, we created a metric of health in the four available waves (2001, 2003, 2012, 2015) of the Mexican Health and Aging Study (MHAS) by employing Bayesian multilevel Item Response Theory. Growth mixture modelling indicated that older Mexicans (n=14,143) age by following four distinct pathways (i.e. high-stable, moderate-stable, low-stable, decliners). Adherence to healthy lifestyle behaviours (i.e. physical activity, non-smoking, limited alcohol consumption) was associated with better health trajectories. Preliminary analyses in the ATHLOS harmonised dataset also suggest that older people age by following four distinct pathways. The impact of lifestyle behaviours within the harmonised dataset will be investigated and also presented.

